# Supplementary material for: Spatially controllable and mechanically switchable isomorphous organoferroeleastic crystal optical waveguides and networks
Source: Nat Commun. 2024 Aug 29;15:7478. doi: 10.1038/s41467-024-51504-5 (PMC11362157; doi:10.1038/s41467-024-51504-5)
Supplement: Supplementary file 1 — Supplementary Information [file 41467_2024_51504_MOESM1_ESM.docx]

Supplementary Information

Spatially Controllable and Mechanically Switchable Isomorphous Organoferroeleastic Crystal Optical Waveguides and Networks

Subham Ranjan^1†^, Avulu Vinod Kumar^2†^, Rajadurai Chandrasekar^2^*, and Satoshi Takamizawa^1^*

^1^Department of Materials System Science, Graduate School of Nanobioscience, Yokohama City University, 22-2 Seto, Kanazawa-ku, Yokohama, Kanagawa 236-0027, Japan

^2^School of Chemistry and Centre for Nanotechnology, University of Hyderabad, Prof. C. R. Rao Road, Gachibowli, Hyderabad 500 046, Telangana (India)
E-mail: [r.chandrasekar@uohyd.ac.in](mailto:r.chandrasekar@uohyd.ac.in), [staka@yokohama-cu.ac.jp](mailto:staka@yokohama-cu.ac.jp)

^†^ Equal contribution of authors

**Table of contents: Page**

| Materials and methods | 3-4 |
| --- | --- |
| Crystallographic and Xpac studies | 4-7 |
| Microscopic studies | 8 |
| 3D-Laser confocal scanning | 9-10 |
| Mechanical studies | 11-13 |
| Crystallographic studies | 13 |
| Solid-state optical properties  Mechanophotonic studies on ferroelastic **1-Cl** crystals  Construction of closed-loop and Z-shaped photonic structures  Mechanophotonic studies on ferroelastic **1-Br** and crystals **1-F**  Fabrication and functioning of hybrid photonic waveguide | 14-15  16  17  18-19  20-21 |
| Surface roughness studies by atomic force microscopy | 21 |
| References | 22 |

**MATERIALS AND METHODS**

**Materials.** 6-bromo-2-hydroxy-3-methoxybenzaldehyde, 4-fluoroaniline, 4-chloroaniline, and 4-bromoaniline were purchased from Tokyo Chemical Industry Co., Ltd. All materials and solvents were of reagent grade and used without additional purification.

**Preparation of Single Crystals.** Equimolar amounts of 6-bromo-2-hydroxy-3-methoxybenzaldehyde and respective anilines (4-fluoroaniline, 4-chloroaniline, and 4-bromoaniline) were dissolved in a 1:1 molar ratio in acetonitrile at 60 °C for around 30 minutes. Slow evaporation yielded block-shaped orange-colored single crystals in about 7 to 10 days.

**Microscopic Observation.** Preliminarily, the mechanical deformation of crystals was investigation by applying force using tweezers and was recorded by an optical microscope (SZ61, Olympus Co.) with inbuilt polarizing plates and a digital camera.

**3D-Laser confocal scanning.** The surface roughness of crystals was determined on the initial and mechanically deformed crystal by a 3D-Laser confocal scanning (LEXT OLS5000, Olympus Co.).

**Force Measurements.**

***Shear test.*** The shear test at room temperature was conducted using a Universal testing machine. A crystal was affixed to a glass base and then sheared using a glass jig attached to a load cell at a displacement rate of 2 µm sec^-1^ on the crystal face (001/00$\bar{1}$). The deformation behavior was observed using a polarized light microscope. The detailed configuration of the measurement can be found in our previous works.^1^

***Three-Point Bending test.*** The three-point bending test experiment was accomplished by using a Universal testing machine coupled with a polarized light microscope. A single crystal was placed on a two-point support and a metal-blade jig was used to apply stress to the crystal. At a displacement rate of 2 µm s^-1^, the jig was pushed on the crystal face (100/$\bar{1}00)$ downward (press). The sharp breaking point was detected after the elastic limit, and the deformation behavior was observed using a polarized light microscope. The detailed configuration and calculation of the measurement can be found in our previous works.^2^

***Single-Crystal X-ray Structure Analysis.*** Single-crystal X-ray diffraction (SCXRD) data of the initial/parent (α_M_) and deformed daughter domain (α_D_) of the obtained **1-X** single crystals were collected and solved based on diffraction patterns on a Bruker D8 VENTURE (PHOTON III 14) using graphite monochromated Mo Kα radiation (λ = 0.71073 Å) at room temperature (rt). Intrinsic phasing methods (SHELXT)^3^ were used to solve the structure, and full-matrix least-squares calculations on F^2^ (SHELXL)^4^ were used to refine it. Bruker (2019) used APEX3 to index the facets of the parent and twinned domains based on the obtained data. Non-hydrogen atoms were refined anisotropically, while hydrogen atoms were fixed at calculated positions using a riding model approximation. Mercury CSD was used to measure Miller plane interplanar angles.

***Solid-State Optical Absorbance and Emission Studies.*** The solid-state optical absorbance spectra were collected using a Shimadzu UV-3600 spectrometer in a diffuse reflectance UV−visible (DR−UV−vis) mode. The solid-state optical emission spectra and the FL quantum yield were measured using the Horiba F1-3C.

***Confocal Optical Microscopy Studies*.** The optical experiments were conducted using a Wi-Tec alpha 200 laser confocal optical microscope facility equipped with a Peltier-cooled CCD detector. A 405 nm continuous wave diode laser was used as an excitation source. The excitation and collection of signals from the output of the crystal waveguides were performed by an upright microscope using 40× and 4× objectives, respectively. The output signal collection was performed using a 4× objective, and the signal was sent to a CCD detector through a multimode optical ﬁber of diameter 100 μm (core). Each spectrum was recorded with an acquisition period of 0.5 s and ten accumulations. All measurements were performed at ambient conditions, and the images were processed by using Wi-Tec Project 5.0 software.

***Scanning Electron Micrographs*.** The size and morphology of the crystals and photonic structures were examined by using a Zeiss field-emission scanning electron microscope (FESEM) operating typically at 6 kV. The crystals and photonic structures were manually transferred from the borosilicate glass substrate to the copper substrate by gently pressing it against the earlier. The gold coating was carried out prior to imaging in all the cases. A few photonic structures were imaged after three months of fabrication to test the stability of deformed crystals.

***Atomic Force Microscope Roughness Measurements.*** The AFM experiments were carried out on an Oxford Asylum Research MFP-3D Origin. The image processing was carried out by using AR 16.25.226 software provided by the manufacturer. The images were recorded in a contact mode topography using a silicon cantilever (AC160TS_R3) with a silicon tip. The dimension of the tip is as follows: cantilever length =100±5 μm, cantilever width =35±3 μm, cantilever thickness =1.7-2.3 μm, resonance frequency =300 (200-400) kHz, force constant =26 (8.4-57 N/m), and tip height =10-20 nm.


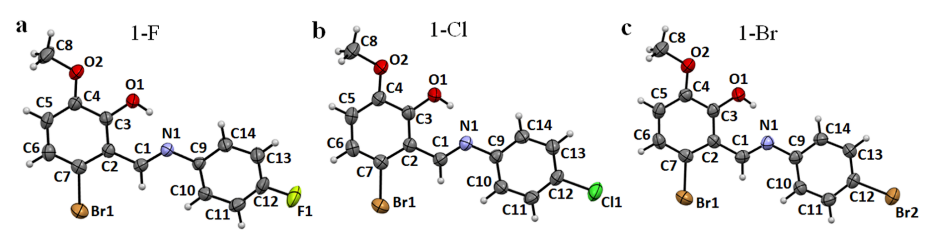


**Supplementary Figure 1. a-c** ORTEP representation of **1-F**, **1-Cl**, and **1-Br**, respectively, where the displacement ellipsoids were drawn at a 50% probability level.

**Supplementary Table 1**. Crystallographic data of mother and daughter domain of crystals **1-Xs**.

| Domain | α_M_ (**1-F**) | α_D_ (**1-F**) | α_M_ (**1-Cl**) | α_D_ (**1-Cl**) | α_M_ (**1-Br**) | α_D_ (**1-Br**) |
| --- | --- | --- | --- | --- | --- | --- |
| T /K | 293(2) | 293(2) | 293(2) | 293(2) | 293(2) | 293(2) |
| Empirical formula | C_14_ H_11_ Br F N O_2_ (Mother Domain) | C_14_ H_11_ Br F N O_2_ (Daughter Domain) | C_14_ H_11_ Br Cl N O_2_ (Mother Domain) | C_14_ H_11_ Br Cl N O_2_ (Daughter Domain) | C_14_ H_11_ Br_2_ N O_2_ (Mother Domain) | C_14_ H_11_ Br_2_ N O_2_ (Daughter Domain) |
| Crystal system | monoclinic | monoclinic | monoclinic | monoclinic | monoclinic | monoclinic |
| Space group | *P*2_1_/*n* | *P*2_1_/*n* | *P*2_1_/*n* | *P*2_1_/*n* | *P*2_1_/*n* | *P*2_1_/*n* |
| *a/Å* | 11.5539(8) | 11.5632(18) | 12.4076(6) | 12.4033(19) | 12.9187(10) | 12.8916(7) |
| *b/Å* | 4.5873(3) | 4.5940(8) | 4.6362(2) | 4.6382(7) | 4.5768(4) | 4.5670(2) |
| *c*/Å | 24.6786(15) | 24.719(4) | 23.6714(11) | 23.678(4) | 23.6168(18) | 23.5707(13) |
| α /° | 90 | 90 | 90 | 90 | 90 | 90 |
| β /° | 92.211(2) | 92.271(5) | 92.075(2) | 92.111(5) | 90.871(3) | 90.982(2) |
| γ /° | 90 | 90 | 90 | 90 | 90 | 90 |
| V /Å3 | 1307.02(15) | 1312.1(4) | 1360.78(11) | 1361.2(4) | 1396.21(19) | 1387.54(12) |
| Z | 4 | 4 | 4 | 4 | 4 | 4 |
| ρ_calcd_ [g cm^–3^] | 1.647 | 1.641 | 1.663 | 1.662 | 1.832 | 1.843 |
| *F*(000) | 648 | 648 | 680 | 680 | 752 | 752 |
| μ [mm^–1^] | 3.153 | 3.141 | 3.213 | 3.212 | 5.802 | 5.838 |
| index ranges | -13 ≤ h ≤ 10,  -4 ≤ k ≤ 5,  -28 ≤ l ≤ 29 | -13 ≤ h ≤ 12,  -4 ≤ k ≤ 5,  -23 ≤ l ≤ 29 | -13 ≤ h ≤ 14,  -5 ≤ k ≤ 5,  -28 ≤ l ≤ 22 | -15 ≤ h ≤ 14,  -5 ≤ k ≤ 5,  -29 ≤ l ≤ 22 | -15 ≤ h ≤ 12,  -5 ≤ k ≤ 4,  -27 ≤ l ≤ 27 | -15 ≤ h ≤ 12,  -5 ≤ k ≤ 5,  -28 ≤ l ≤ 25 |
| Reflections collected | 2313 | 2282 | 2406 | 2716 | 2458 | 2455 |
| Goodness of fit | 1.085 | 1.212 | 1.181 | 1.026 | 1.180 | 1.156 |
| *R*_1_  (*I*> 2σ (all data)) | 0.0354 | 0.0522 | 0.0305 | 0.0673 | 0.0391 | 0.0390 |
| w*R*_2_  (*I*> 2σ (all data)) | 0.1384 | 0.1627 | 0.01134 | 0.1890 | 0.1039 | 0.1161 |
| CCDC No. | 2308107 | 2308108 | 2308109 | 2308110 | 2308111 | 2308112 |

**Supplementary Table 2**. Summary and comparison of unit cell parameters and unit cell similarity index of **1-F**, **1-Cl**, and **1-Br** crystals.

| **Derivative Pair** | **Unit Cell Parameters** | | | **Unit Cell Similarity Index** |
| --- | --- | --- | --- | --- |
| **1-F** & **1-Cl** | a=11.5539  b=4.5873  c=24.6786 | α=90  β=92.211  γ=90 | Volume: 1307.02 | 0.002569 |
|  | a=12.4076  b=4.6362  c=23.6714 | α=90  β=92.075  γ=90 | Volume: 1360.78 |  |
| **1-Cl** & **1-Br** | a=12.4076  b=4.6362  c=23.6714 | α=90  β=92.075  γ=90 | Volume: 1360.78 | 0.00965 |
|  | a=12.9187  b=4.5768  c=23.6168 | α=90  β=90.871  γ=90 | Volume: 1396.21 |  |
| **1-Br** & **1-F** | a=12.9187  b=4.5768  c=23.6168 | α=90  β=90.871  γ=90 | Volume: 1396.21 | 0.00716 |
|  | a=11.5539  b=4.5873  c=24.6786 | α=90  β=92.211  γ=90 | Volume: 1307.02 |  |

The unit-cell properties of two crystal structures are used to calculate the unit-cell similarity index (π), which was used to decode the isostructurality quantitatively. The value of 'π' should be near zero if the compared structures are generally isostructural; if it is zero, the structures are isomorphous.^5^ In this instance, the value of 'π' to each other of the **1-F**, **1-Cl**, and **1-Br** is close to zero (to the second decimal place; see Table S2), illustrating the pairs' isomorphous nature.


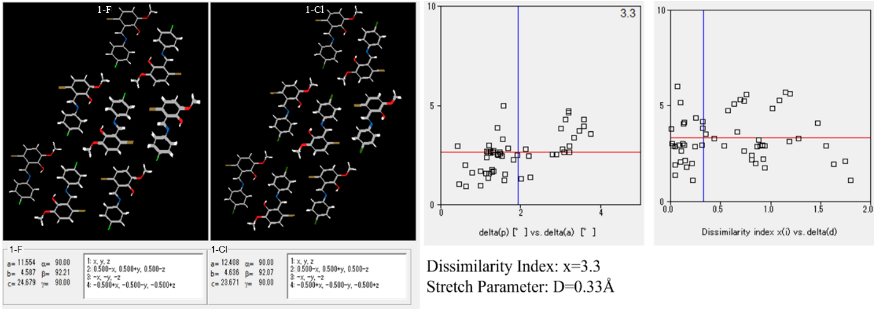


**Supplementary Figure 2**. 3D supramolecular constructs between **1-F** and **1-Cl** deduced from XPac analysis.


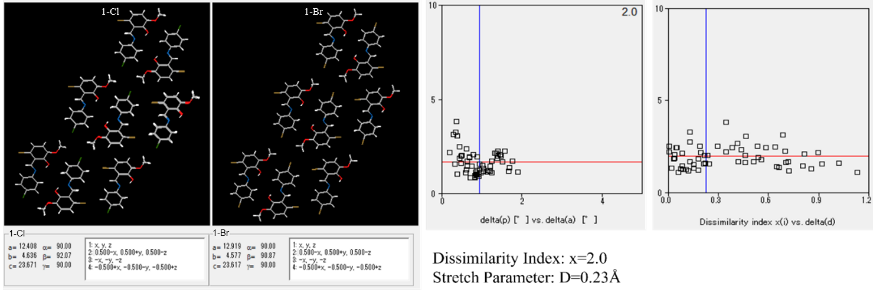


**Supplementary Figure 3**. 3D supramolecular constructs between **1-Cl** and **1-Br** deduced from XPac analysis.


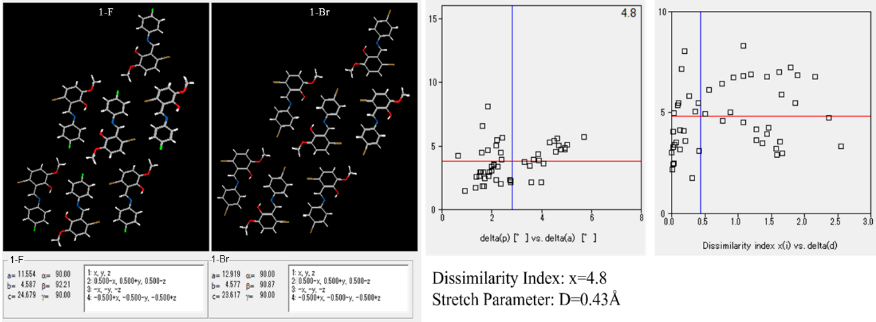


**Supplementary Figure 4**. 3D supramolecular constructs between **1-F** and **1-Br** deduced from XPac analysis.


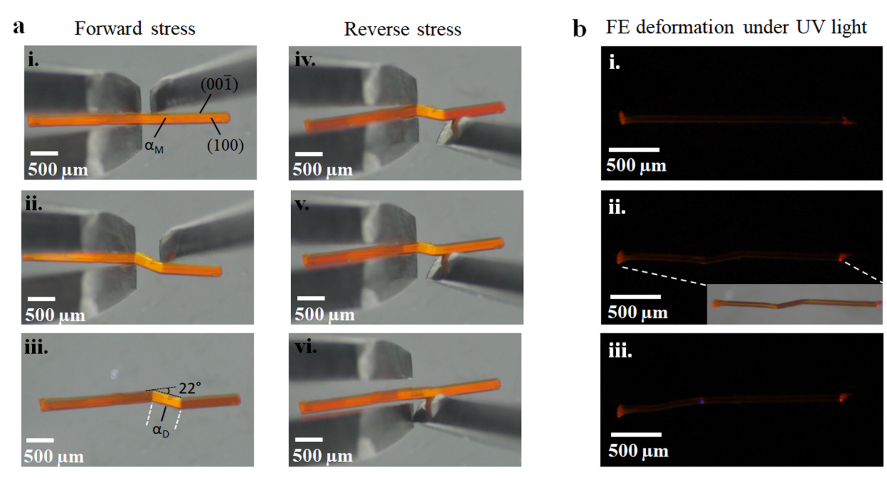


**Supplementary Figure 5.** Snapshots of ferroelastic deformation of single crystal **1-Cl** under **a** polarized white light (PWL) and **b** UV light (365 nm). Inset in **b, ii** shows optical image under PWL.
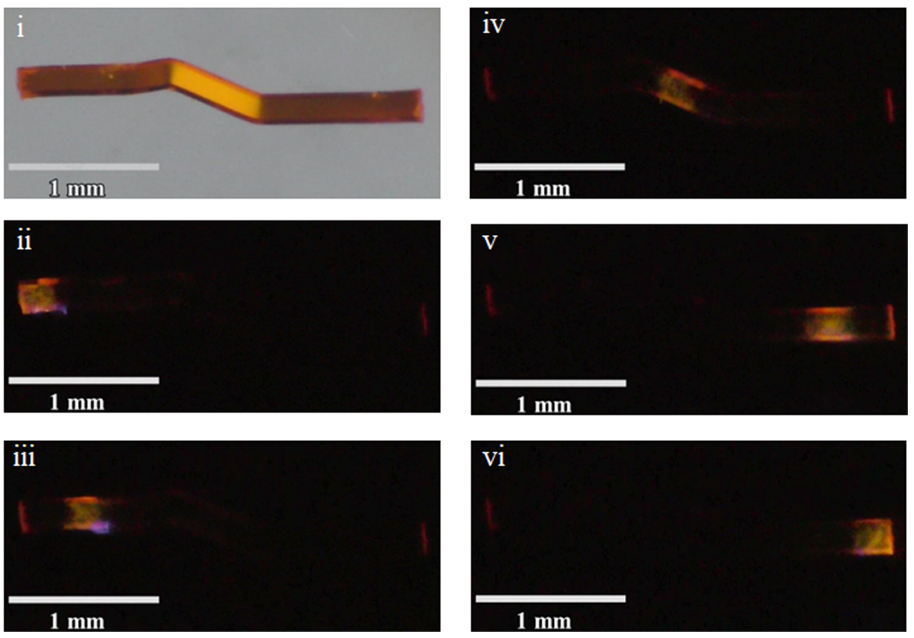


**Supplementary Figure 6. i-vi** Snapshots of light guiding ability of ferroelastically deformed single crystal **1-Cl**. An in-house fabricated micron-sized 405 nm laser was used for preliminary investigation of waveguiding property. The light was irradiated from the top of the crystals.

**
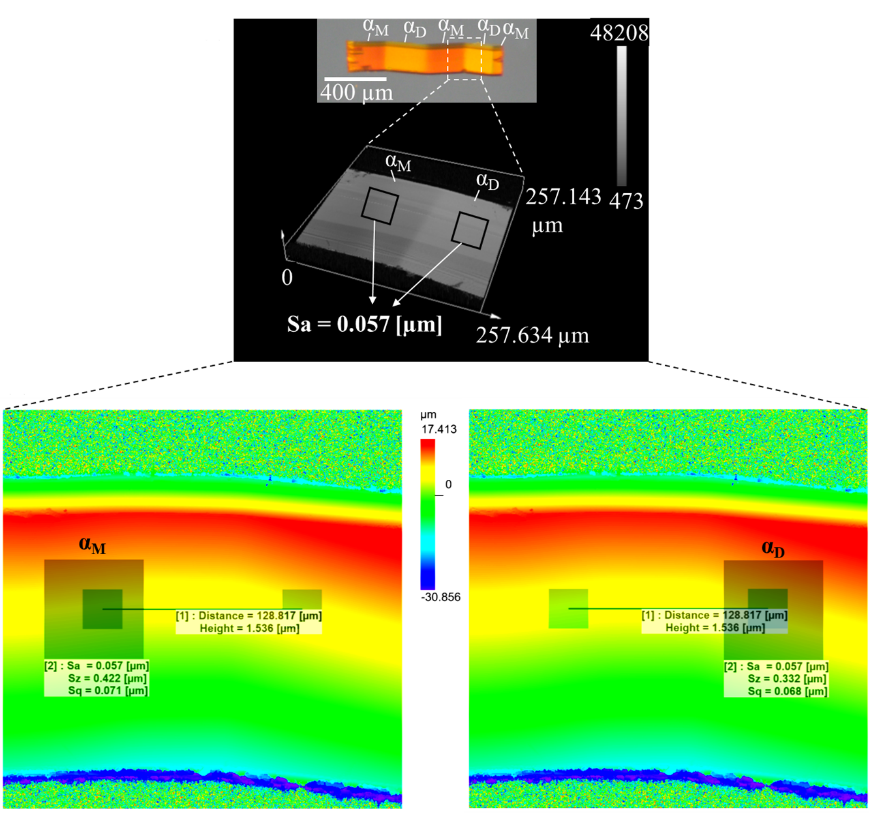
**

**Supplementary Figure 7.** Snapshot of ferroelastically deformed crystal (**1-Cl**), enlarged part of **1-Cl** crystal studied for surface roughness evaluation applied to roughness standard. The areal roughness value *S*a (≈0.057 μm) determined from measurement was similar in the case of the mother and daughter domain. The surface roughness of the mother and daughter domain can be easily perceived and distinguished due to the contrast imaging.


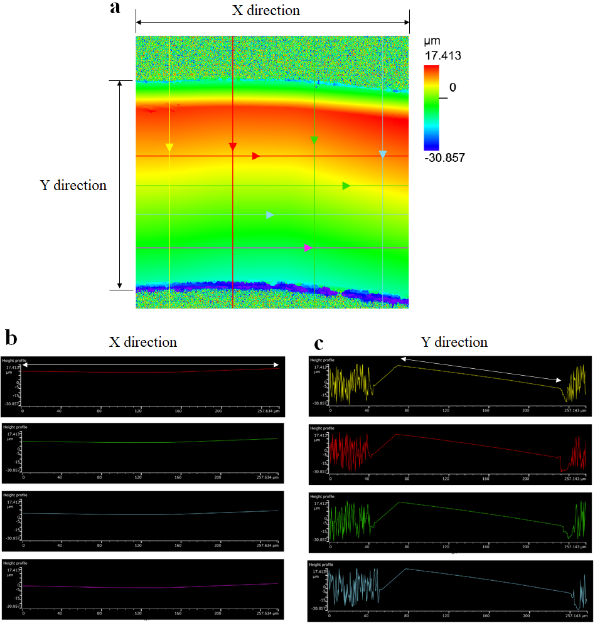


**Supplementary Figure 8. a** Results of 1-D roughness measurement of ferroelastically deformed crystal of **1-Cl** illustrate the roughness features in **b** X direction and **c** Y direction. The bi-directional arrow indicates the length of the X and Y direction of the size of the crystal.


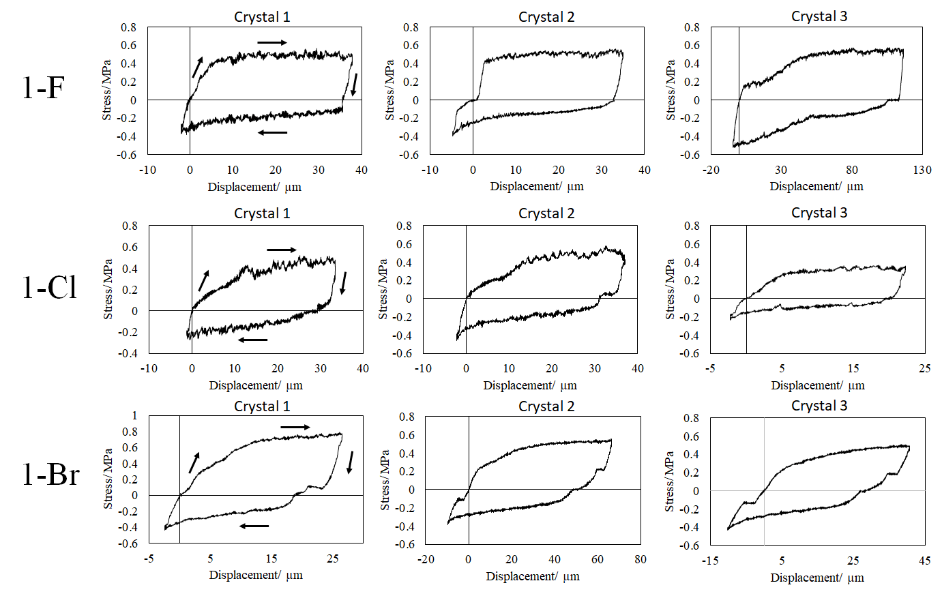


**Supplementary Figure 9.** Stress-displacement curves of **1-F**, **1-Cl**, and **1-Br** during the shear test on the (00$\bar{1}) \mathrm{plane}$.


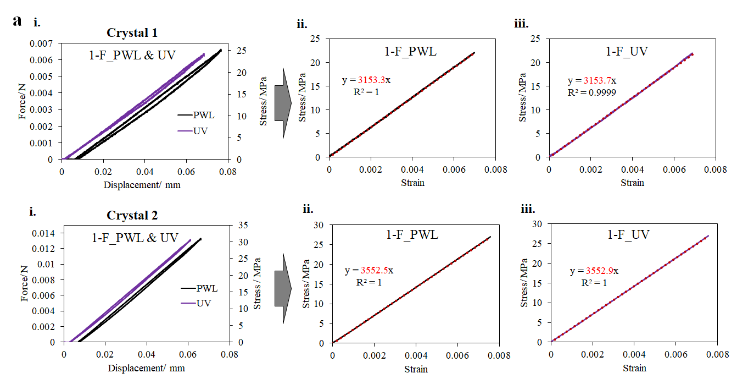


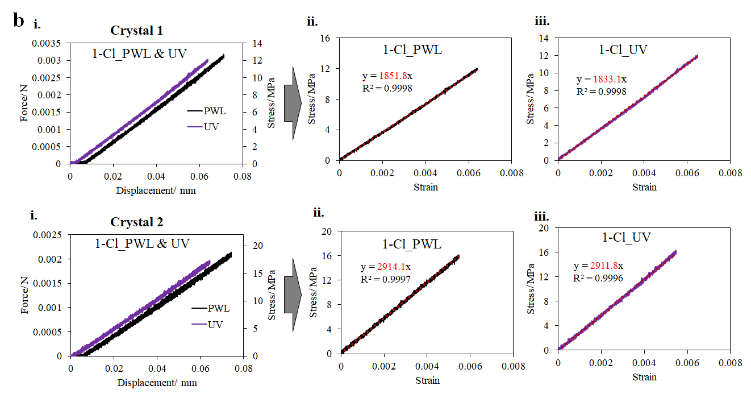

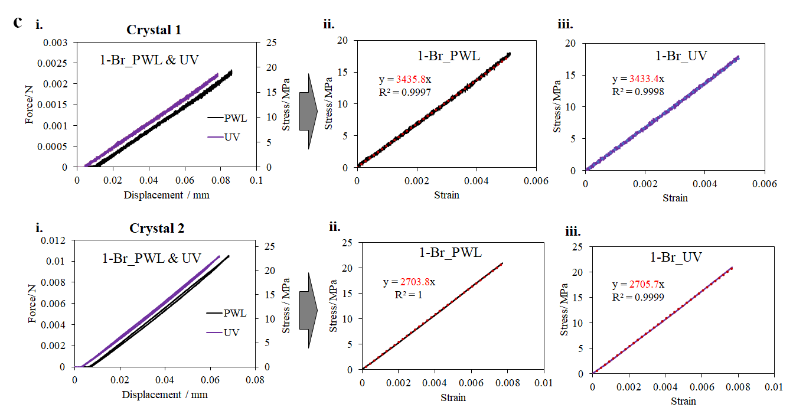


**Supplementary Figure 10. a-c** Stress-strain curves of **1-F**, **1-Cl,** and **1-Br** during the three-point bending test, respectively. The slope of stress-strain graphs gives the calculated elastic moduli.


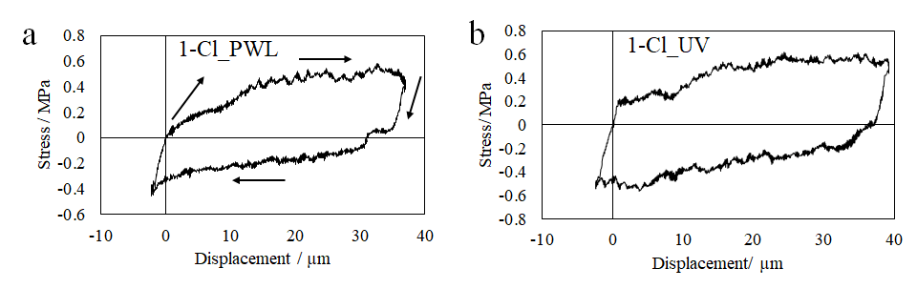


**Supplementary Figure 11.** Stress-displacement curves of **1-Cl** under **a** PWL and **b** UV.


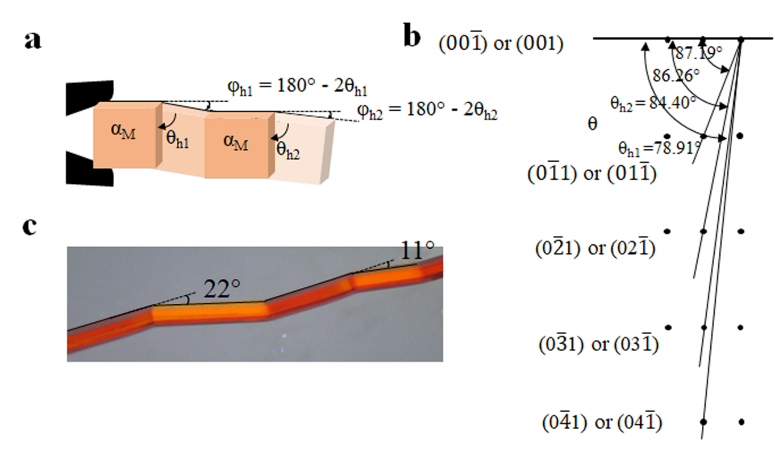


**Supplementary Figure 12. a** A relationship between θ and a bending angle of φ. **b** Calculated angles θ for several different twin interfaces. and **c** Photographs of a bent crystal of **1**-**Cl** with varying bending angles, such as 22° and 11°.


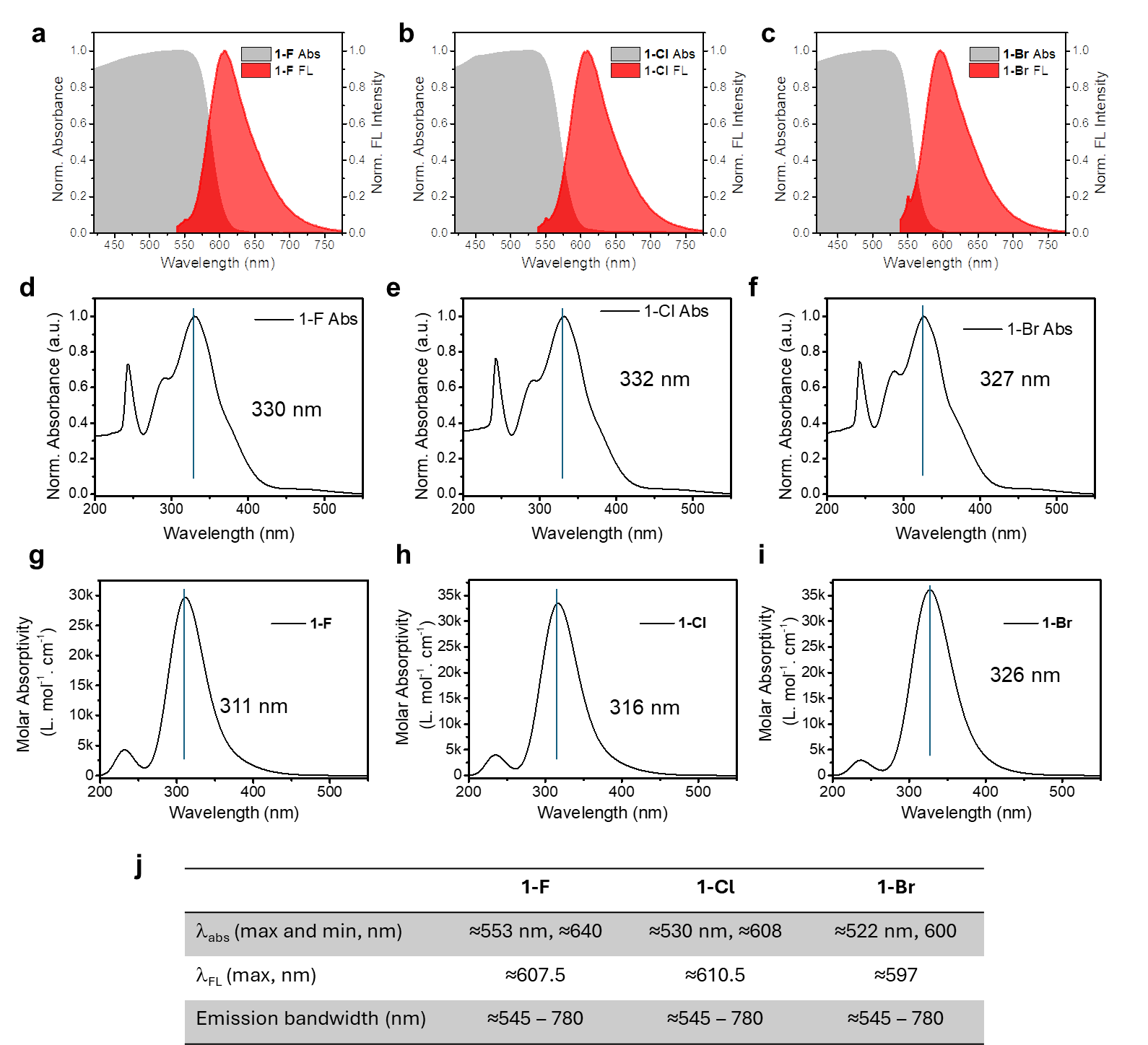


**Supplementary Figure 13. a-c** Solid-state optical absorption and emission spectra of **1-F**, **1-Cl**, and **1-Br**, respectively. **d-f** Solution-state absorption spectra. **g-i** The calculated electronic properties using the TD-DFT method with functional B3LYP and 6−311+G(d,p) basis sets and of **1-F, 1-Cl** and **1-Br**, respectively. TD-DFT computations are quite comparable to experimental results, and they help to provide insight to support the studies.

**Table 3.** The solid state optical absorption maximum, absorption tail minimum, fluorescence (FL) maximum and FL spectral bandwidth of **1-F**, **1-Cl**, and **1-Br**, respectively.


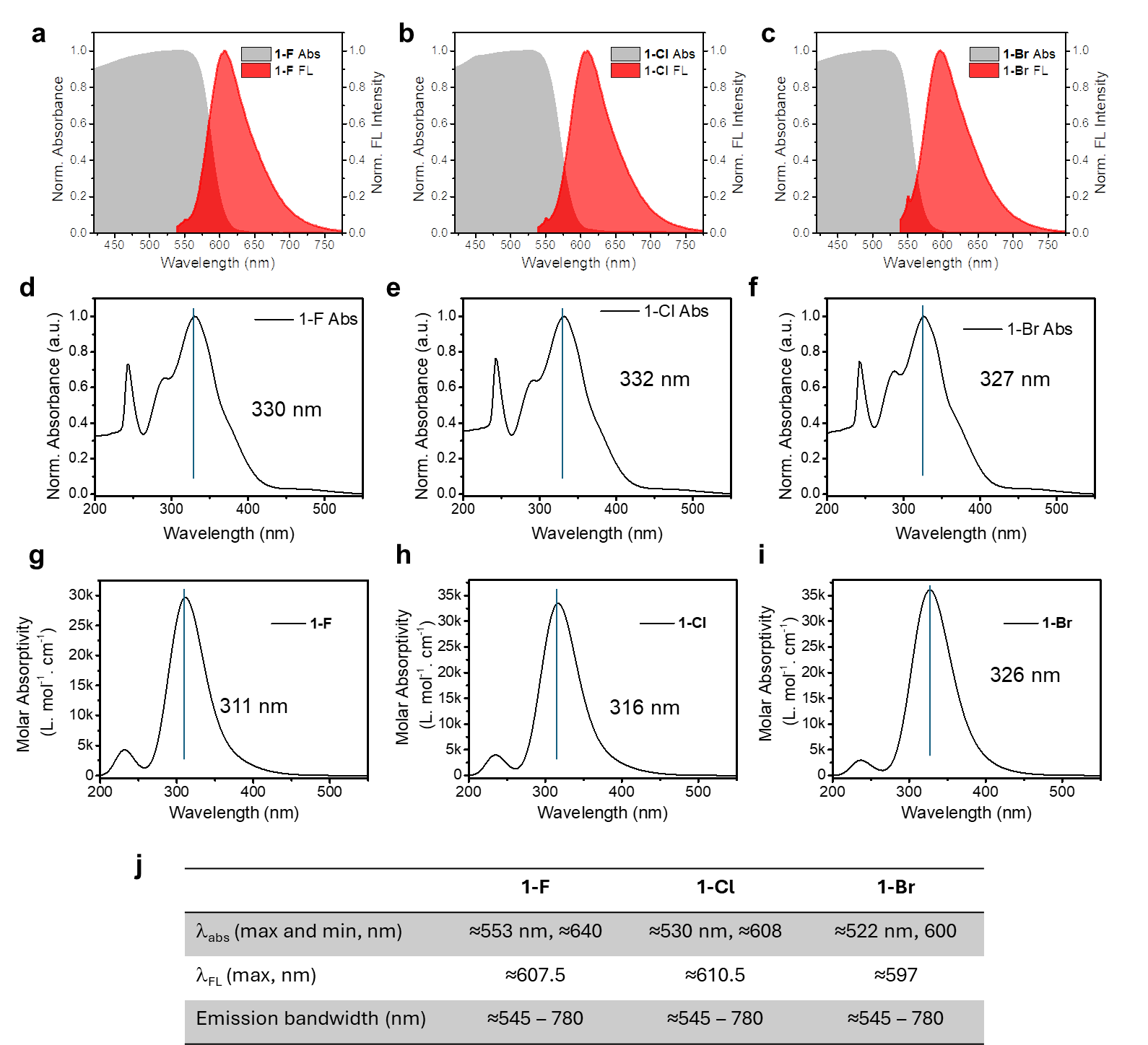


**Supplementary Notes:**

**Optical loss calculation**:

The optical loss of the waveguides was calculated using the following equation

${I_{\mathrm{tip}}}/{I_{\mathrm{body}}}$ = $e^{-\alpha\mathbf{D}}$ (1)

where $I_{\mathrm{tip}}$ corresponds to FL intensities at collection terminal of the waveguide and$I_{\mathrm{body}}$ is the FL intensity at the excitation terminal (in this case, the left terminal) of the waveguide. **D** is the distance or optical path length travelled by the light between excitation (left terminal and body) and collection positions.

Optical loss coefficient,^6^

α′ (dB cm^-1^) = α(cm^-1^) × 4.343 (2)

**Supplementary Figure 14.** Optical loss of **1-Cl** crystal WG, before and after ferroelastic bending, calculated using the fit from I_tip_/I_body_ vs distance between laser excitation point and detector position (light propagation distance).


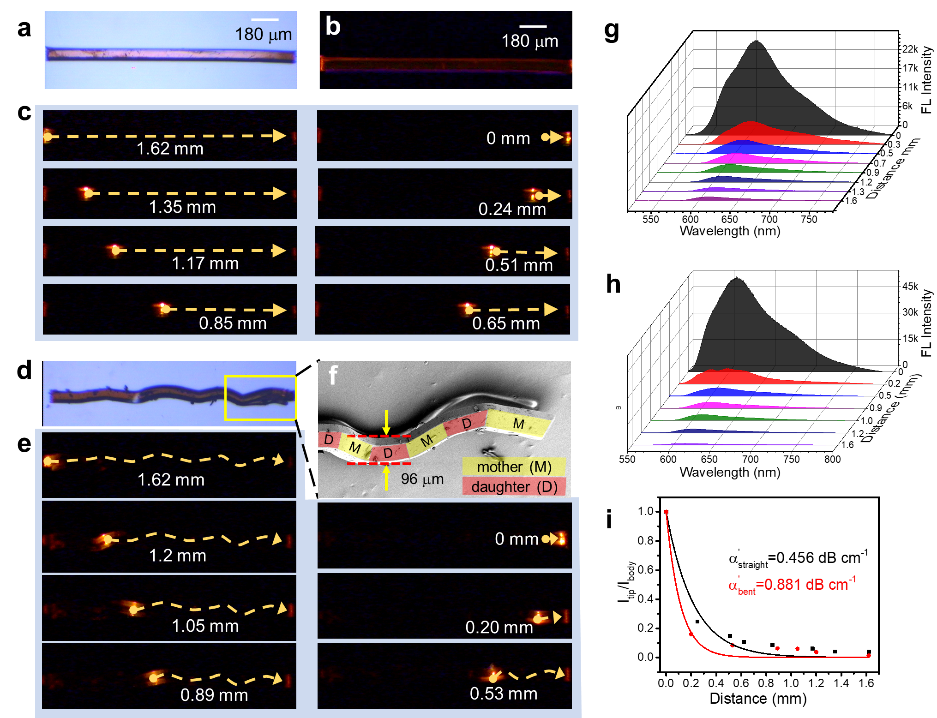


**Supplementary Figure 15.** **a** Confocal optical, **b** FL (crystal excited with a UV torch) and **c** FL (crystal excited with a laser), microscope images of straight **1-Cl** crystal waveguide for different excitation positions. **d** Confocal optical and **e** FL (crystal excited with a laser), microscope images of ferroelastic bent **1-Cl** crystal waveguide. **f** Colour-coded FESEM image of a ferroelastic bent **1-Cl** crystal waveguide. **g,h** Laser excitation position-dependent light guiding studies performed on a straight and ferroelastic bent **1-Cl** crystal waveguide, respectively. **i** Optical loss observed before and after ferroelastic bending in **1-Cl** crystal waveguide.


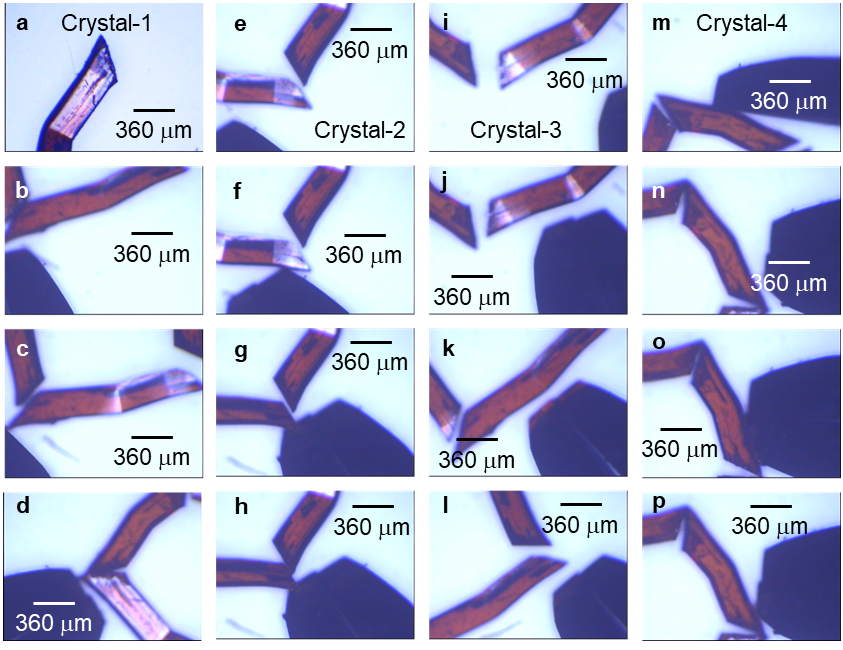


**Supplementary Figure 16.** **a-p** Optical microscope images illustrating the mechanical integration of ferroelastic bent **1-Cl** crystal waveguides into a closed-loop structure using tweezers where **a** and **p** indicate the beginning and final stage of the integration of ferroelastic bent **1-Cl** crystals, respectively.


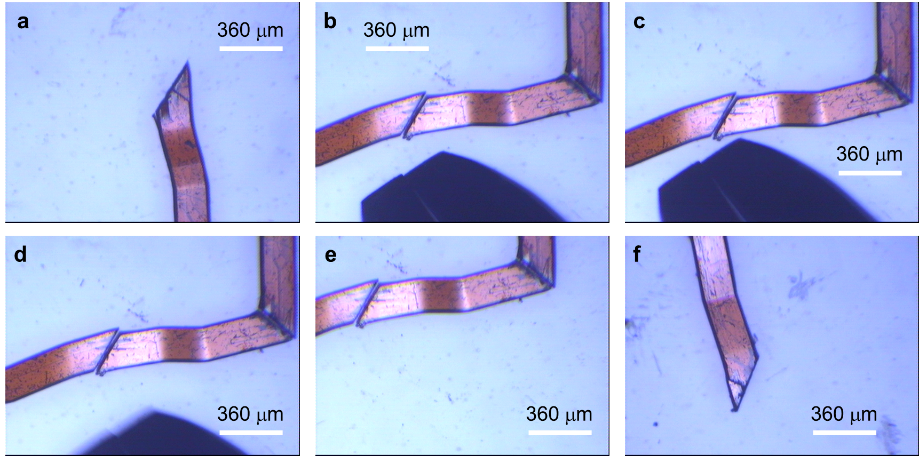


**Supplementary Figure 17.** **a-f** Optical microscope images depicting the mechanical positioning of ferroelastic bent **1-Cl** crystal waveguides into a Z-shaped photonic structure using tweezers.


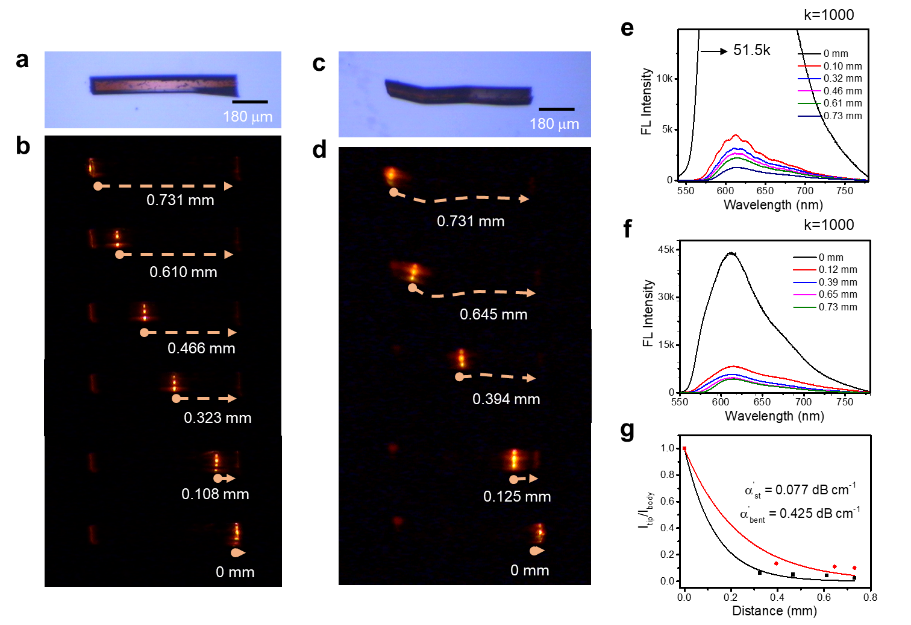


**Supplementary Figure 18.** **a** Confocal optical and **b** FL microscope (crystal excited with a laser) images of straight **1-Br** crystal waveguide for different excitation positions. **c** Confocal optical and **d** FL microscope images of ferroelastic bent **1-Br** crystal waveguide. **e**,**f** Laser excitation position-dependent light guiding studies performed on a straight and ferroelastic bent **1-Br** crystal waveguide, respectively. **g** Optical loss observed before and after ferroelastic bending in **1-Br** crystal waveguide.


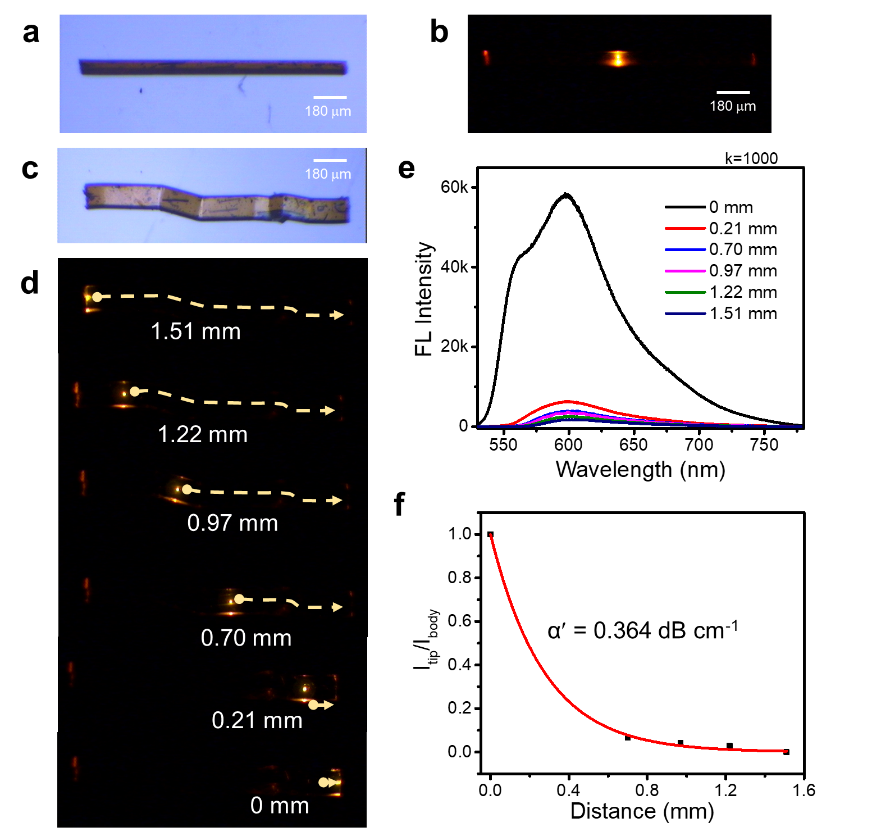


**Supplementary Figure 19.** **a** Confocal optical and **b** FL microscope (crystal excited with a laser) images of straight **1-F** crystal waveguide for different excitation positions. **c** Confocal optical and **d** FL microscope images of ferroelastic bent **1-F** crystal waveguide. The crystal in **a** and **c** are viewed along thinner and thicker facets, respectively. **e** Laser excitation position-dependent emission spectra recorded on a **1-F** ferroelastic bent crystal waveguide. **f** Optical loss estimated for the **1-F** ferroelastic bent crystal waveguide.


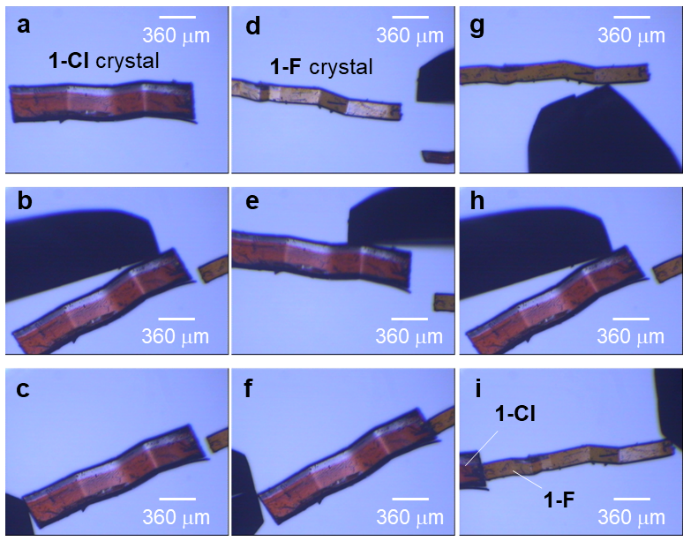


**Supplementary Figure 20.** **a-i** Mechanical integration of **1-Cl** with **1-F**. Confocal optical images depicting the terminal-to-terminal mechanical integration of ferroelastic bent **1-Cl** and **1-F** crystals using tweezers.


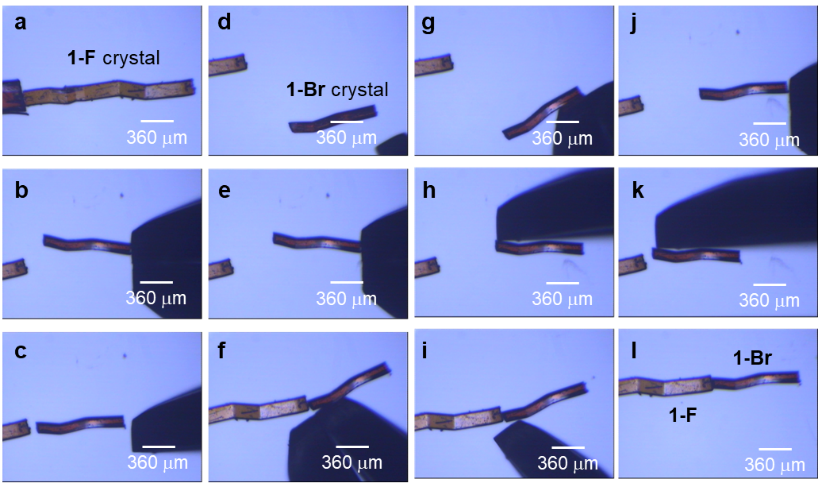


**Supplementary Figure 21.** **a-l** Mechanical integration of **1-Br** to previously coupled **1-F** and **1-Cl**. Confocal optical images showing the fabrication of hybrid photonic waveguide by positioning **1-Cl**, **1-F** and **1-Br** crystals in a terminal-to-terminal fashion.


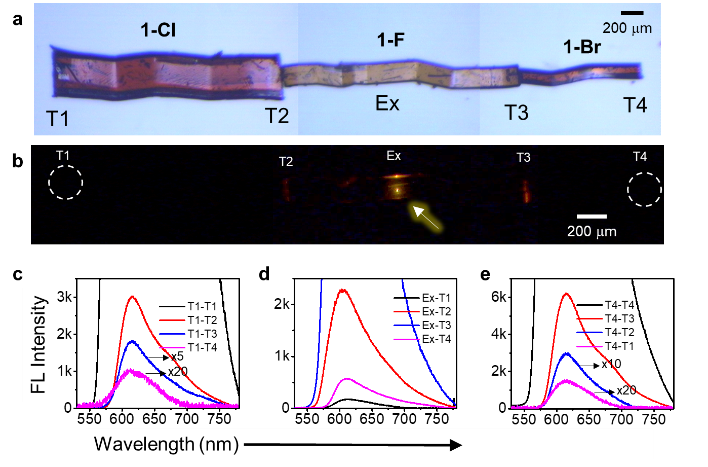


**Supplementary Figure 22.** **a** Stiched Confocal optical and **b** FL microscope images of hybrid photonic waveguide, respectively. **c-e** FL spectra recorded at various termini in hybrid photonic waveguide for laser light input at T1, Ex, and T1, respectively. All experiments were performed using 4 mW power.


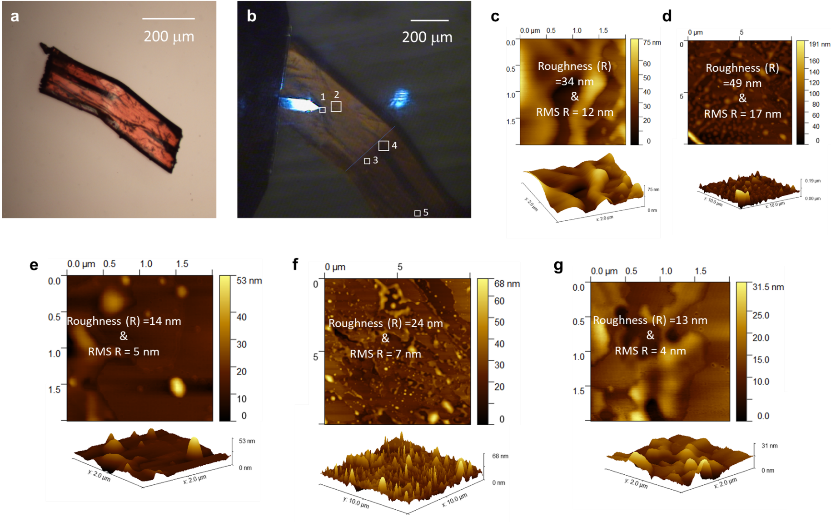


**Supplementary Figure 23. a** Snapshot of unevenly ferroelastically deformed crystal (**1-Cl**), **b** Analysed area for surface roughness, **c-g** Surface topography from area #1 to #5, respectively. and h surface and root mean square roughness of area #1**-**#5.

**Supplementary** **Notes:** The surface roughness values obtained for the unevenly bent crystal are lower than those reported for other optical waveguides.^7^

**Supplementary References**:

1 Mir, S. H., Takasaki, Y., Engel, E. R. & Takamizawa, S. Ferroelasticity in an organic crystal: a macroscopic and molecular level study. *Angew. Chem. Int. Ed.* **56**, 15882-15885 (2017).

2 Ranjan, S. *et al.* Structural and Thermal Diffusivity Analysis of an Organoferroelastic Crystal Showing Scissor-Like Two-Directional Deformation Induced by Uniaxial Compression. *J. Am. Chem. Soc.* **145**, 23027-23036 (2023).

3 Sheldrick, G. M. SHELXT–Integrated space-group and crystal-structure determination. *Acta Crystallogr. A: Found. Adv.* **71**, 3-8 (2015).

4 Sheldrick, G. M. Crystal structure refinement with SHELXL. *Acta Crystallogr. C Struct. Chem.* **71**, 3-8 (2015).

5 Ranjan, S. *et al.* Isomorphism:molecular similarity to crystal structure similarity'in multicomponent forms of analgesic drugs tolfenamic and mefenamic acid. *IUCrJ* **7**, 173-183 (2020).

6 Quimby, R. S. *Photonics and lasers – An Introduction*, John Wiley & Sons, Hoboken,

New Jersey (2006).

7 Sun, D., Shang, H., & Hiang, H. Effective metrology and standard of the surface roughness of micro/nanoscale waveguides with confocal laser scanning microscopy. *Opt. Lett.* **44**, 747-750 (2019).
