# Supplementary material for: Spatially controllable and mechanically switchable isomorphous organoferroeleastic crystal optical waveguides and networks
Source: Nat Commun. 2024 Aug 29;15:7478. doi: 10.1038/s41467-024-51504-5 (PMC11362157; doi:10.1038/s41467-024-51504-5)
Supplement: Supplementary file 3 — Description of Additional Supplementary Files [file 41467_2024_51504_MOESM3_ESM.pdf]

Description of Additional Supplementary Files

File Name: Supplementary Movie 1
Description: Shear stress-induced mechanical deformation on $(00\bar{1})$ plane of crystal **1-Cl** by a pair of tweezers under polarized white light (PWL).

File Name: Supplementary Movie 2
Description: Shear stress-induced mechanical deformation on $(00\bar{1})$ plane of crystal **1-Cl** by a pair of tweezers under UV light (365 nm).

File Name: Supplementary Movie 3
Description: Preliminary investigation of light propagation on ferroelastically deformed 1-Cl crystal under micron-sized 405 nm laser light.

File Name: Supplementary Movie 4
Description: Shear stress-induced mechanical deformation on $(00\bar{1})$ plane of crystal **1-Cl** under PWL during force measurement.

File Name: Supplementary Movie 5
Description: Shear stress-induced mechanical deformation on $(00\bar{1})$ plane of crystal **1-Cl 2** under UV during force measurement.

File Name: Supplementary Movie 6
Description: Three-point stress-induced mechanical deformation on $(100/\bar{1}00)$ plane of crystal **1-Cl** under PWL till breaking point.

File Name: Supplementary Movie 7
Description: Three-point stress-induced mechanical deformation on $(100/\bar{1}00)$ plane of crystal **1-Cl** under PWL and UV.

File Name: Supplementary Movie 8
Description: A demonstration of a long crystal cut by a razor blade with an approximate angle of 45°.
